# Regenerative Medicine and Angiogenesis; Focused on Cardiovascular Disease

**DOI:** 10.34172/apb.2022.072

**Published:** 2021-10-03

**Authors:** Seyed Zachariah Moradi, Faramarz Jalili, Zohreh Hoseinkhani, Kamran Mansouri

**Affiliations:** ^1^Pharmaceutical Sciences Research Center, Health Institute, Kermanshah University of Medical Sciences, Kermanshah, Iran.; ^2^Medical Biology Research Center, Kermanshah University of Medical Sciences, Kermanshah, Iran.; ^3^Gradute Studies Student, Sobey School of Business, Saint Mary‚S University, Halifax, NS,Canada.; ^4^Molecular Medicine Department, Faculty of Medicine, Kermanshah University of Medical Sciences, Kermanshah, Iran.

**Keywords:** Cardiovascular disease, Angiogenesis, Regenerative medicine, Stem cell therapy, Gene therapy, Tissue engineering, VEGF

## Abstract

Cardiovascular disease (CVD) is a major concern for health with high mortality rates around the world. CVD is often associated with partial or full occlusion of the blood vessel network. Changes in lifestyle can be useful for management early-stage disease but in the advanced stage, surgical interventions or pharmacological are needed to increase the blood flow through the affected tissue or to reduce the energy requirements. Regeneration medicine is a new science that has provided many different options for treating various diseases, especially in CVD over the years. Stem cell therapy, gene therapy, and tissue engineering are some of the powerful branches of the field that have given patients great hope in improving their condition. In this review, we attempted to examine the beneficial effects, challenges, and contradictory effects of angiogenesis in vivo, and in vitro models’ studies of CVD. We hope that this information will be able to help other researchers to design new effective structures and open new avenues for the treatment of CVD with the help of angiogenesis and regeneration medicine in the future.

## Introduction

###  Regenerative medicine, current strategies, and clinical applications

“Regenerative medicine” is a term coined by Leland Kaiser in 1992 and 7 years later, once again was used by William Haseltine in the Lake Como conference in 1999. Regenerative medicine is a scientist that efforts to regenerate and replace damaged human organs, tissues, and cells, and try to restore normal functioning and activities to a damaged body. It seems that this technology may provide new hope for the treatment of various diseases.^1,2^

One of the relatively common cases of body parts regeneration observed in nature is the reconstruction and regeneration of the salamanders cut the limb, within a few days, it can reconstruct the amputated limb.^3^ Similarly, humans up to 11 years have such an ability to regenerate the separated fingertip. Although humans have lost this ability over time, it seems that this potential for regeneration remains slightly.^4^

 There are considerably three variant approaches to obtain regenerative medicine purposes:

cell-based therapy, material science, and bioengineering (or in other words implantation of scaffolds seeded with cells) that this third strategy is a combination of the two previous approaches.^1,5^

In the history of regenerative medicine, for the first time in 1992, the bladder obtained from the laboratory was used for patients with myelomeningocele. The artificial bladder showed good effects and acceptable results.^6^ 10 years later, a liver tissue obtained from the laboratory as the first solid organ introduced, in vitro studies of new tissue showed defensible and appropriate results and evince good effects in studied rats.^7^ Today regeneration medicine has notably advanced and has acquired a remarkable ability to regenerate different organs and tissues of the body accordingly, many researchers around the world are working to prepare and advance the achievements of this field and make it suitable for use in clinical. For example in 2015 for treatment of retinal disease, Human embryonic stem (HESCs) cells were used to improve the conditions of nine patients with dry age-related macular degeneration (AMD) and nine patients with Stargardt’s macular dystrophy. The results were good and 10 of 18 patients showed considerable improvement in visual acuity. After 22 months follow-up of patients treated with HESCs, evidence showed that HESCs was safe and well-tolerated, furthermore, any proof for rejection, adverse proliferation, drastic systemic or ocular complication was not observed.^8^ In another study, the impact, performance, and safety of human embryonic stem cells (HESCs)-derived cardiovascular progenitors in six patients with severe ischemic left ventricular dysfunction was studied. The influence of HESCs to generate clinical-grade cardiovascular progenitor cells was investigated and results were acceptable.^9^ Skin tissue is also one of the most well-known organs that many groups around the world have been trying to redesign or improve on existing specimens. Today, different brands such as Dermagraft®, Apligraf®, OrCel®, etc have been marketed by different companies for wound healing or burns.^10^ The spectrum of diseases that may be improved and treated by HESCs based therapies as a branch of regeneration medicine is enhancing, and several clinical trials for investigating the effect of ES-derived cell products on type 1 diabetes mellitus, amyotrophic lateral sclerosis and spinal cord injury started.^11^

###  Cardiovascular disease (CVD)

The subcategory diseases of CVD include all heart diseases and circulatory system such as peripheral arterial disease, coronary heart disease (CHD), venous thromboembolism, cerebrovascular disease, congenital, and rheumatic heart diseases. According to the World Health Organization (WHO) report, CVD is the primary death cause worldwide. Based on the report, CVD is the most prevalent ones. Approximately about 31% of all deaths worldwide in 2016 (approximately 17.9 million) were due to CVD.^12,13^ According to projections, by 2030 more than 23.3 million people will die from CVD annually, though CVD incidence decreased in many developed countries. The main causes of this may be because of higher exposition to cardiovascular risk factors, prevention program’s shortage, and insufficient access to equitable and effective healthcare services. CVD is also known to be between 15 leading conditions causing extensive functional disability, and it affects life quality and the ability to work. It is predicted that the disability resulting from CVD, being 85 million in 1990, rise to 150 million in 2020.^14^ influential important risk factors of CVD and its development include physical inactivity, tobacco smoking, abdominal obesity, heavy use of alcohol, and unhealthy diet. To observe and consider these risk factors can prevent CVD significantly and the related beneficial effects are demonstrated.^12,13^ Because CVD patients significantly risk following CVD events, including myocardial infarction (MI), stroke, and death, therapeutic lifestyle variations and change such as enhancing physical activity, weight loss, improving diet quality and health (dietary correction), and stop smoking is recommended for all these patients. Furthermore, drug therapies, especially aspirin and statins, may be an adjunct in most high-risk patients. Besides, since the adverse and complications of cardiovascular outcomes may be increased by influenza-like infections, influenza vaccination may improve and reduce the outcome of these diseases.^15^ Despite new successes in CVD patients’ treatment options, many patients with advanced are still there and due to various co-morbidities prohibiting them from undergoing surgical procedures, surgical revascularization is impossible for them.^16^

###  Angiogenesis and its importance

The term “angiogenesis” is used to describe the physiological process of blood vessel growth. Angiogenesis is essential for the growth and regeneration of organs and tissues. In the significant number of disorders that occur such as infectious, malignant, ischemic, inflammatory, and immune disorders there is an imbalance in this process, for this reason, research on angiogenesis, its inducers, inhibitors, and its influencing factors, can have significant effects on the treatment of some number of diseases.^16^ In adults, angiogenesis takes place only during physiological healing processes such as wound healing, cycling ovary, furthermore, during pregnancy angiogenesis occurs in the placenta. This physiological process is regulated by inducing and inhibiting factors, and the balance between these two groups that if this balance breaks down, significant potential for disorders such as tumors and malignancies will be provided.^16,17^ However endothelial-cells preserve their ability to generate new vessels and by altering the conditions and the appearance of physiological stimulators, such as hypoxia and inflammation for blood vessels and lymph vessels respectively, these cells become capable and expressing their ability.^16^ Hypoxic tissue and target sites produce and release angiogenic factors such as Ang2, vascular endothelial growth factor (VEGF), basic fibroblast growth factor (bFGF), and promote the early-stage of angiogenesis. In the following, the expression of tyrosine kinases receptors like Tie-1 and 2 and VEGFR-2 on surfaces of the endothelial cell will up-regulate and the intracellular signaling pathways promote. After binding pro-angiogenic factors to their receptors on endothelial cells, they lead to the activation of these cells. The endothelial cell activation is known with the high mitotic index, increasing the capacity for the invasion, and matrix proteolysis. Activated endothelial cells can invade the connection between cells of the basement membrane and extracellular matrix, and disrupt gap junctions between endothelial cells. Furthermore, with the inception of endothelial cell activity, certain types of matrix metalloproteinases (MMPs) secrete from these cells and facilitate the breakdown of the basement membrane in the region. In the later stages of the angiogenesis process, MMPs are produced to break down the extracellular matrix and innate its regeneration, by digesting the basement membrane, endothelial cells migrate and proliferate, also, binding molecules such as integrin αvβ3 and αvβ5 contribute to facilitating the process of pulling, advancing, and give direction the buds of growing blood vessels then the process of tube formation begins with the interaction of Ang-Tie-2, this complex assistance the stabilizing vessel structure. In the next step, the EphB-ephrinB system also regulates the process of tubular formation, and eventually, pericytes and smooth muscle cells are added to stabilize the newly formed blood vessel.^18-20^

**Figure 1:**
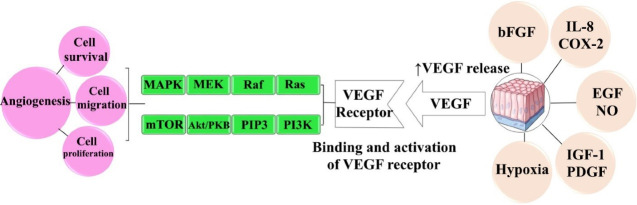
The stimulus factors and signaling pathways involved in the angiogenesis process

Although the inhibition of angiogenesis in combination with conventional chemotherapy is used as one of the therapeutic strategies in the treatment of cancer, however, angiogenesis may be one of the significant therapeutic targets in patients with the cardiovascular disorder such as myocardial ischemia, coronary artery disease, and either poor or abnormal vascularization.^21,22^ The stimulus factors and signaling pathways involved in the angiogenesis process are summarized in Figure 1.

###  Recent advances in therapeutic angiogenesis

The VEGF, FGF, MMP, Delta-like ligand 4 (DII4), angiopoietins (Ang) and class 3 semaphorins (SEMA3s) are some of the most well-known compounds that can stimulate angiogenesis.^23-25^ Several studies have attempted to investigate the notable effects of these compounds on angiogenesis and the potential of these compounds and their similar compounds in the treatment of related diseases. For example, in another study, indirect vasoconstrictive surgery accompanied by administration of plasmid human vascular endothelial growth factor gene (phVEGF) could notedly enhanced the capillary density in the brain of the rat models. This approach may be a suitable therapy in patients suffering from chronic cerebral hypoperfusion.^26^ In 2016 Chade et al to improve the bioavailability and pharmacokinetics of VEGF planned a study. They designed and synthesized a stabilized biopolymer, in this new structure the elastin-like polypeptide (ELP) was fused with VEGF protein and its potential and efficacy for therapeutic renal angiogenesis in swine models of renovascular disease (RVD) was studied. In summary renal microvascular (MV) density restored and fibrogenic activity subtilized. Also, improvements in the rate of glomerular filtration rate and renal blood flow were deduced. Furthermore, the level expression of VEGF and its receptor was improved. They concluded that ELP-VEGF therapy can help in RVD models and these beneficial effects may be directed via lessening the activity of fibrogenic and restoration of the signaling of renal angiogenic.^27^ In another study, the effects of VEGF gene therapy on the intensification of sciatic nerve regeneration was investigated. Results emphasize that in the animals that were treated with VEGF, the numbers of blood vessels and myelinated fibers notably higher than others. Furthermore, the sciatic functional index and survival of neuron cell bodies improved. The authors believe that these good results are related to the positive effects of VEGF and its potent ability to stimulate angiogenesis and its neuroprotective effects.^28^ To examine new strategies for the treatment of chronic wounds and burns in humans, Pereira et al investigated the effects of mesenchymal stromal cells (MSCs) and human bone morphogenic protein-2 (hBMP-2) on animal models. The results showed that simultaneous injection of MSCs and hBMP-2 at the wound site could synergistically increase angiogenesis and micro-vessel formation as well as increase proliferation or migration of keratinocytes and fibroblasts.^29^

## Role of regenerative medicine in angiogenesis

###  Stem cells potential for angiogenesis

**Figure 2:**
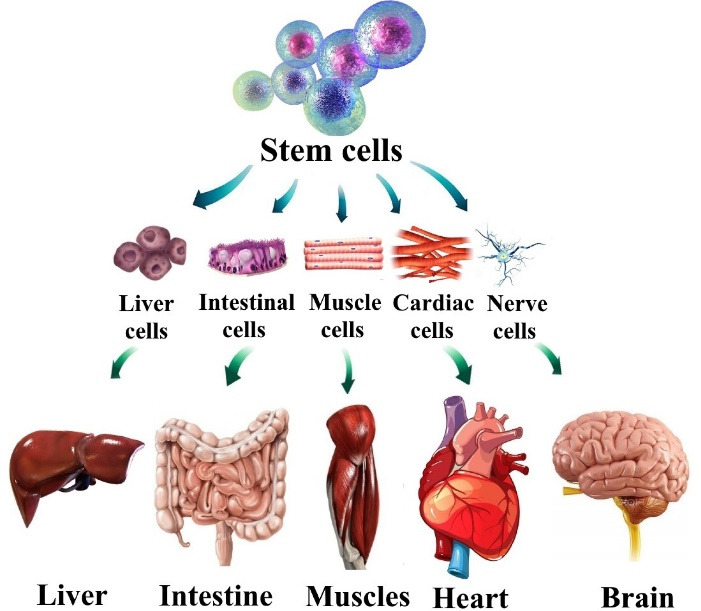
The potential application of human stem cells

New blood vessel’s growth sprouting from pre-existing vessels is known as angiogenesis. In the process, endothelial cells are proliferated and activated by stimulation and migrated towards the angiogenic stimuli. Afterward, the small vessels are formed and in larger vessels, perivascular supporting and mural cells such as pericytes and smooth muscle cells surround endothelial cells. Furthermore, non-vascular cell types such as stromal cells and bone marrow-derived hematopoietic cells are an important contributor to angiogenesis by producing various factors that promote de novo blood vessel’s growth and expansion.^30^ Stem cells have clonogenic and self-renewing capabilities and are a subclass of undifferentiated cells (Figure 2). They are found in peripheral blood, bone marrow, cardiac tissue, adipose tissue, muscle tissue, etc, and differentiate into specialized cell types under certain conditions. For initiation of the angiogenesis process directly (by differentiated daughter cell’s production) or indirectly (by progeny production producing paracrine growth factors) stem cells are necessary.^31^

According to Rinkevich et al, the endothelial stem/progenitor cells involved in adult angiogenesis must be local, non-hematopoietic, and non-circulating tissue-resident cells. Also, some evidence show that there is a rare population of vascular endothelial stem cell capable of producing very high endothelial daughter cells number is the main factor for adult’s neovascular growth. Stem and progenitor cell subsets and also vascular cell types or other perivascular, including smooth muscle cells or pericytes, directly contribute to neovascular growth. Tissue-infiltrating hematopoietic cells that are produced by hematopoietic stem cells by remodeling the extracellular matrix or in a paracrine, manner contributes to angiogenesis. During recent years, evidence indicated that progenitor cell and hematopoietic stem subpopulations in various tissues were an angiogenesis requirement and is a good idea for novel approaches development and therapeutic aims for therapies and antiangiogenic treatments for cardiovascular repair.^32-36^

Research suggests that cardiac stem cells improve myocardial function recovery after ischemia. These cells have a good ability to differentiate to cardiomyocytes to replace damaged cells and the activation of signal transducer and activator of transcription 3 (STAT3) in the myocardia by stromal cell-derived factor-1 (SDF-1), a chemokine that is secreted by cardiac stem cells. Inhibition of SDF-1 secretion blocked recovery. SDF-1 recruits stem cells to the infarcted heart and result in the improvement of cardiomyocytes’ survival by decreasing caspase 3-dependent apoptosis. Furthermore, cardiac stem cells, MSCs are capable of doing this function. They, just as cardiac stem cells, induce STAT3 phosphorylation in the myocardia. Also, MSCs may lead to cardiomyocytes enhanced survival through a paracrine mechanism. It was indicated that conditioned media from MSCs exposed to hypoxia resulted in the cytoprotective effect of isolated adult rat ventricular cardiomyocytes and reduced the infarct size in a rodent infarct model after MSC transplantation meaningfully. It was especially reported that conditioned media from MSCs overexpressing the Akt gene (Akt-MSCs) inhibit isolated cardiomyocytes apoptosis exposed to hypoxia through caspase 3 attenuation releases and ends in morphologic and apoptotic cell death. Furthermore, Akt-MSC released paracrine factors that control injured myocardium mediating protection. Moreover, in comparison with control MSCs, Sfrp2 is upregulated in Akt- MSCs, and its diminishing by siRNA silencing abrogated Akt-MSC mediated cytoprotective activities. Hypoxic, in response to hypoxia, induced Akt regulated stem cell factor (HASF), upregulate in Akt-MSCs, and can mediate survival effects in isolated hypoxic cardiomyocytes through interfering with the PKC-ε signaling pathway that may result in cardioprotection by activation blocking of mitochondrial death channels.^37-39^

###  Chemical stimulators and their potential for angiogenesis

In recent years, numerous studies have been accomplished to identify the new factors and constituents that may be effective in angiogenesis, which has led to the discovery of multiple compounds and the mechanisms of their efficacy in the process of angiogenesis. Chemical stimulators play a vital role in the process of angiogenesis. This category is composed of various compounds, this seems that among the influential factors, likely VEGF is the most essential and important factor for the differentiation and development of the vascular system so that the result of losing a single VEGF allele in embryonic is lethal.^40^ Some of the most important chemical stimulators and their major functions are shown in Table 1. On the other hand, indiscriminate and unnecessary increased angiogenesis can cause various problems such as hemangiomas, Kaposi’s sarcoma, Ocular neovascularization, etc, so that, the therapeutic target of a notable number of anticancer drugs is the inhibition of angiogenesis. For this reason, a balance between angiogenesis-inducing factors and its inhibitory factors is crucial.^41^

**Table 1:** Some of the most important chemical stimulators and their major functions

**Stimulator**	**Major functions**	**References**
VEGF family	Stimulator of angiogenesis and lymphangiogenesis.	^42,43^
Fibroblast growth factor family	Regulates the proliferation, differentiation, and migration of endothelial cells	^42^
Angiopoietin 1 and 2 (Ang1, Ang2)	Stimulates angiogenesis, stabilize vessels and regulate cell growth and division	^25,44^
Transforming growth factor-alpha (TGF-𝛼)	Induction of extracellular matrix production	^45^
Cyclooxygenase-2 (COX-2)	Induction of angiogenesis and vasodilation	^25,46^
Platelet-derived growth factor (PDGF)	Angiogenesis stimulation and regulate the division and growth of cells.	^47,48^
Integrins αvβ3, αvβ5, α5β1	Receptors for proteinases and matrix macromolecules	^25^
Ephrins	Determine and specification the formation of veins or arteries - Stimulates the production of extracellular matrix and endothelial cell proliferation	^49,50^
Hepatocyte growth factor (HGF)	Stimulates cell growth	^50,51^
Plasminogen activators	Releases and activates growth factors, remodels extracellular matrix	^25^
Interleukin 8	Incite proliferation and survival of the endothelial cell and increase the production of MMP	^50,52^
Id1/Id3	Determine endothelial plasticity	^25^

In many studies, scientists have attempted to exploit the notable potential of this effective and pivotal tool to inspire and stimulate angiogenesis in human or animal studies. For example, becaplermin, a recombinant human platelet-derived growth factor-BB (PDGF-BB), is the first FDA-approved drug for the angiogenesis approach in 1977. The drug’s trade name is Regranex®, and that was released in the pharmaceutical form of Gel. Regranex® was indicated for the treatment of diabetic foot ulcer disease. Although it’s effective, for the sake of high cost and number of postmarketing reports, Regranex® use has been limited. Some postmarketing reports suggested that in comparison with the control group, patients were treated with three or more tubes of Regranex®, which showed the enhanced rate of mortality secondary to malignancy.^53^ In 2019 Melly et al attempted to stimulate cardiac angiogenesis via growth factors. They designed a fibrin gel delivery platform to help the co-delivery of VEGF and PDGF-BB. Optimized fibrin platform is used to strict control duration and the distribution of growth factor dose in the myocardium. Result suggests that in the short period of treatment, the optimized platform could stimulate angiogenesis.^54^ To improve the angiogenesis, functional, and neurogenesis properties in ischemia rats models, Sugiura et al investigated the advantages of intraventricular injection of a recombinant adenovirus-expressing heparin-binding epidermal growth factor-like growth factor (HB-EGF). The results suggest that gene therapy using Ad-HB-EGF could help the functional recovery after ischemic stroke this effect becomes practical through promoting angiogenesis and neurogenesis.^55^ In 2001 the protection effects of VEGF to reduce renal fibrosis and improving the function of renal was investigated in remnant kidney models animal. In summary, VEGF could stabilize renal function and depauperate renal scarring that it seems this effect duo to a partial reversal of the impaired angiogenesis. The results of this study showed that the use of angiogenic factors such as VEGF or other compounds may be effective for the treatment of progressive renal disease.^56^ Furthermore, Wang et al designed a new system of the vasoactive intestinal peptide, this team loaded its microspheres in the polycaprolactone nanofibrous membrane and investigated its effect on wound healing and angiogenesis in animals model. They found that the new system could notedly improve the angiogenesis and wound healing in the studied animals and it may be a suitable option in wound treatment and reliable point for vascular tissue engineering.^57^ The delivery results of VEGF, PDGF, and FGF-2 from the polymeric system could increase the *in vitro* angiogenesis differentiation of human umbilical vein endothelial cells (HUVECs), besides, the formation of mature vascular networks accelerated in the chorioallantoic membrane. Briefly, advantages emphasize that delivery of VEGF, FGF-2, and PDGF equals considerable angiogenesis.^58^

###  Tissue engineering and angiogenesis

Tissue engineering is an interdisciplinary field among biology, medicine, and engineering. It was originally conceptualized about 25 years ago and is a growing field and. It deals with biomaterials, employs cells, and bioactive moieties like growth factors.^59^ Tissue engineering generally aims at replacing, repairing, and regenerating damaged organs and tissues. Usually, it is achieved by a combination of cells with highly porous scaffold biomaterials operating as formats and templates for tissue regeneration to control and guide new tissue growth. Microvascular network’s promotion inside engineered tissue constructs requires angiogenesis.^60^ In clinical studies, the engineered tissues need an efficient vascular network to supply and safeguard the cell’s oxygen and nutrients. A proper vascular network before newly engineered tissue implantation can help and support in this regard by connecting to the patient’s vasculature. However, by supplying and covering all cells with sufficient nutrients and oxygen, the production shall be well organized.^61^ The primary conformation and organization of vascular networks can be controlled by microfabrication technology such as bioprinting and photopatterning and also, adapting the local microenvironment controls the geometry of vascular networks. The fluid flows, mechanical signals patterning, and growth factors are considered as vascular organization important factors.^61^ if tissue engineering goal is to replace large tissue portions, understanding of the exact mechanisms and factors regulating the angiogenic process is necessary. Therefore, tissue engineering research focuses on angiogenesis analysis. The angiogenesis process is a rare one in adults and limited to cancer and post-injury regeneration. These angiogenesis types have outcomes that are very different. Interconnected and functional vessels are generated in reparative angiogenesis, while a high number of disorganized and immature vessels exist in cancer. Today, a large number of *in vitro* and *in vivo* assays and animal angiogenesis models have been developed. Furthermore, preclinical angiogenesis assays have employed.^62^ Two of the *in vivo* model assays (the dorsal skinfold chamber and the chorioallantoic membrane) recently have been more commonly employed in tissue engineering research.^63^ The chicken chorioallantoic membrane is an extraembryonic membrane highly vascularized that is used for researching angiogenesis, metastasis, and cancer invasion. It is an attractive preclinical *in vivo* model for studies of vascular growth and/or for drug screening.^64^ Application of dorsal skinfold chamber models makes *in vivo* vascularization pathophysiological studies in a continuous period. It is a model ideal to research angiogenesis when intravital continuous measurements are necessary and allow diverse tissues and textures analysis. This model’s long history indicated its value in angiogenesis assessment.^65^

###  The effects of gene therapy on angiogenesis

Gene therapy is an experimental technique that involves the transfer of genetic material to treat or prevent disease. It is a very powerful tool to improve or control the disease process and predicted that in the future, gene therapy allows doctors to treat many disorders via inserting a gene into a patient’s cells instead of using drugs or surgery.^66,67^ Depending on the disorders and their pathology, we may expect different effects of gene therapy. For example, in disorders that gene is overexpressed, maybe gene knockdown be helpful, and in contrast, disorders that occur due to a loss of function of a gene, it is expected that gene therapy will be effective and improves the status of the patient.^67^ Gene therapy is one of the most powerful tools available to researchers that can play an important role in angiogenesis. To date, several drugs with similar mechanisms have received approval from the U.S. Food and Drug Administration (FDA) to treat various diseases. Table 2 summarizes some of the drugs in this category and their indications.

**Table 2:** Some of the FDA approved drugs with gene therapies based mechanism

**Drug name**	**Brand name**	**Disorder**	**Reference**
Nusinersen	Spinraza	Spinal muscular atrophy (SMA)	^68^
Tisagenlecleucel	Kymriah	Diffuse large B-cell lymphoma Acute lymphoblastic leukemia	^69^
Voretigene neparvovec	Luxturna	Retinal dystrophy	^70^
Onasemnogene abeparvovec	Zolgensma	Spinal muscular atrophy (SMA)	^71^
Axicabtagene ciloleucel	Yescarta	Large B-cell lymphoma	^72^
Talimogene laherparepvec	Imlygic	Melanoma	^73^
Alipogene tiparvovec	Glybera	lipoprotein lipase deficiency (LPLD)	^74^

In many studies, gene therapy has been helpful for angiogenesis and it was one of the important tools for controlling angiogenesis. In 2005 Sugiura et al investigated the beneficial effects of recombinant adenovirus-expressing heparin-binding epidermal growth factor-like growth factor (Ad-HB-EGF) on neurogenesis, angiogenesis, and functional consequence after focal cerebral ischemia in Wistar rats. Result emphasize that Ad-HB-EGF can contribute to functional recovery via elevating angiogenesis and neurogenesis in animals model of ischemic stroke.^75^ In another study, a new protein transduction domain (Hph-1-GAL4; ARVRRRGPRR) was used to overexpress the hypoxia-inducible factor-1 alpha (HIF1A) and investigated it’s in vitro and in vivo effects on angiogenesis. The matrigel plug assay used for identifying the angiogenic response and in vitro results showed that each matrigel plug was evaluated and the expression levels of HIF1A, and HIF1A target genes were notedly higher than in control. Furthermore, blood vessels were observed in the group treated with HIF1A in vivo study and expression levels of VEGF and Cd31 were remarkably higher than the control group.^76^ Multicistronic lentivectors expressing SERCA2a (contractile function), apelin (cardioprotection), and FGF 2 (angiogenesis) designed and injected into the myocardial tissue of the mouse model of MI. Heart function parameters, prevention of fibrosis, and the number of the vessel were improved.^77^ In 2011 Pereira et al investigated the beneficial effect of VEGF gene therapy on sciatic nerve regeneration. They found that VEGF gene therapy can increase the sciatic nerve regeneration and the number of blood vessels in VEGF-treated animals than the control group.^29^ Besides, after investigation the effects of intramuscular injection of plasmid coding VEGF165 (pVEGF) on animal models of myocardial ischemia, the human studies were performed. The results of animal studies indicated that the produced system can induce cardiomyogenesis and arteriogenesis in animals. No significant effects were observed. For this reason the Phase I trial to evaluate the efficacy and safety of pVEGF gene transfer in patients with severe coronary artery disease that were not inclined to formal revascularization (PCI and/or CABG). During 6 months follow up symptoms and ejection fraction (EF)% were improved and myocardial ischemia was reduced.^78^ In the same way, Kusumanto et al try to characterize the effect of intramuscular administration of VEGF gene-carrying plasmid (phVEGF165) on in 54 adult diabetic patients with critical limb ischemia. Compared with the placebo group, patients treated with a VEGF165-containing plasmid showed considerable and remarkable improvement. Also, no significant side effects were observed.^79^ The effect of intramyocardial VEGF-2 gene transfer on 30 patients with intractable Canadian cardiovascular society class III or IV angina was investigated. After 1 year follow-up, the angina symptoms in the greater part of patients during the first year of treatment were improved.^80^ In another study, the effect of combination delivery of HGF and VEGF165 genes to reduce the consequences in the Wistar rats model of MI investigated. In summary, the results confirmed the regulation of inflammatory response in addition to the increase in capillary density.^81^

## Appropriate effects of angiogenesis on CVD

As previously discussed, CVD is often related to partial or full blood vessel network occlusion. Therapeutic angiogenesis induction may be an appropriate strategy for CVDs treatment. At the time being, treatment options available in CVDs have focused on re-establish blood flow through the vascular beds affected according to the disease stage. Therapeutic angiogenesis is employed by new blood vessels grow from pre-existing vessels for re-supply blood flow to affected tissues. Two common strategies are by delivery of stem/progenitor cells or growth factors.^82^

Conventionally, therapeutic angiogenesis has roots in growth factors-controlled application, helping to initiate angiogenesis in target tissues. Recent developments in angiogenic growth factor’s recognition in therapeutic angiogenesis largely promoted the CVDs treatment. Various clinical and animal studies acknowledged them as important treatment modalities for CVDs.^83^ Some most important factors of angiogenic growth including VEGFs, FGFs, and PDGF can be delivered as target proteins or genes. VEGFs (in particular, VEGF-A) are involved both in arteriogenesis and in capillary growth, induced by tissue ischemia. Five members of the VEGF-A and VEGF family is the member most studied.^84^ VEGF therapy was evaluated in research administering the VEGF protein, and in gene therapy trials that VEGF gene was administered in a viral or plasmid vector. KAT, VIVA, NORTHERN, and Euroinject One Trial are the most important trials performed with VEGF.

KAT trial - Kuopio Angiogenesis Trial, a placebo-controlled, randomized, double-blind phase II study, assessed the feasibility and safety of catheter-based local intracoronary VEGF gene transfer in the prevention of in-stent restenosis and post angioplasty and the treatment of chronic myocardial ischemia. Patients with CHD were recruited in this study. Local VEGF gene transfer offered during angioplasty was conducted by standard methods and then by gene transfer along with a perfusion-infusion catheter. VEGF plasmid liposome and VEGF adenovirus were administered to patients, and ringer’s lactate to control patients. The follow-up time was 6 months. The data obtained indicated that intracoronary gene transfer can be conducted with safety, no differences in minimal lumen diameter or clinical restenosis rate existed, and an obvious increase was observed in myocardial perfusion in patients treated with VEGF-adenovirus.^85^

Euroinject One trial - Euroinject One phase II, randomized double-blind trial, assessed the therapeutic angiogenesis of percutaneous intramyocardial plasmid gene transfer of VEGF (phVEGF-A(165)) on myocardial perfusion, clinical symptoms, and left ventricular function. Patients suffering from severe stable ischemic heart disease were randomly assigned to administer placebo plasmid or phVEGF-A(165) in the myocardial region indicating stress-induced myocardial perfusion defaults on (99m) Tc sestamibi/tetrofosmin single-photon emission computed tomography. Compared with placebo, the VEGF gene transfer didn’t significantly result in enhanced stress-induced myocardial perfusion abnormalities; although, enhanced regional wall motion, evaluated by ventriculography and NOGA, may show a favorable anti-ischemic effect.^86^

NORTHERN Trial – in the refractory class 3 or 4 angina trial patients were assigned randomly to injection VEGF-A placebo or plasmid. No difference was observed in placebo-treated or VEGF-treated patients after three- or six-months regarding SPECT-evaluated ischemic burden. It is interesting that both groups showed a significant reduction in a time-dependent manner in the ischemic burden. No clinical studies exist about the efficacy of the VEGF family other members, including PlGF and VEGF-B.^87^

Prolonged exposure of tissue to angiogenic factors is necessary for the survival of newly formed vasculature and angiogenesis. Because of low stability, rapid diffusion, and angiogenic factors shot half-lives, multiple injections or excessive doses are needed that results in unstable vessel growth and uncontrolled vascular formation in undesired locations. To improve the angiogenic factors short half-life and slow diffusion from target sites, biomaterials can be used as systems of drug delivery. They may improve therapeutic efficiency in intramyocardial injection therapy. Biomaterial injections in combination with the biological-controlled release improve cardiac regeneration as a result of sustaining biomolecule delivery, localizing material, extending their half-live, and protecting the biologic factors. In this biomaterial, biochemical and electrostatic interactions are used to control release. The thermal gels are used to offer temperature-responsive properties for the drugs-controlled release and can be injected clinically with a robotic injection system for intramyocardial injection therapy in a minimally invasive manner. Results of an in vivo research indicated that the thermal gel for the localized, sustained VEGF release with the intramyocardial injection can be employed. Therefore, the thermal gel intramyocardial injection possesses vascularization properties for CVDs treatment.^88^

Cell therapy is used for functionally impaired tissue regeneration in organs. In induced pluripotent stem cells, stem cell therapy embryonic stem cells, and adult stem cells including mesenchymal stem cells and hematopoietic stem cells may be employed. In angiogenesis, it serves by delivering cells into the growing vascular supply by building blocks so as to form new blood vessels. The cell type ideal for successful CVDs cell-based therapy should possess the following characteristics: the ability to transdifferentiate into mature cell types as a response to microenvironmental stimuli, high plasticity, cellular and angiogenesis survival promotion through paracrine mediators’ secretion, low carcinogenicity and immunogenicity, high resilience in hypoxic microenvironments, anti-inflammatory functions, and simple cultivation and generation. Besides political and ethical concerns, Pluripotent stem cells involve teratoma formation risk. The problem mentioned is also related to induced pluripotent stem cells. Mesenchymal stem cells are the cells most qualified for therapeutic purposes. Their main defects include poorly low survival rates and transdifferentiated following transplantation into ischemic areas. But angiogenic and anti-inflammatory factors paracrine secretion cause their positive effects. The cells deriving from Monocyte are also appropriate for cell-based therapy. An *in vitro* research indicated that peripheral blood monocytes increased their plasticity state and expression of pluripotency various markers following growth factor-induced reprogramming. These cells are known as programmable cells of monocytic origin (PCMO). PCMO can induce in vitro angiogenesis via proangiogenic factors release Their Transplantation into mice chronic ischemic hind limbs improved tissue oxygenation and induced neovascularization. The regulatory macrophage is another cell type suitable for cell-based CVD therapies. Results obtained from a study acknowledged using them as a treatment option based on cell therapy-based for CVDs.90 another therapeutic angiogenesis strategy is Transmyocardial laser revascularization, a new surgical indirect revascularization technique acting via host angiogenic response stimulation through injuring the ischemic myocardium via laser ablation in specified locations.^82,89^

Chinese herbal medicines are effective in CVDs treatment extremely employed in many countries, especially in China since ancient times, however, the mechanisms through which herbal medicine prevents and treats CVDs are still not known. They indicated the pro-angiogenic properties with an unclear mechanism. They have some effects on endothelial cell’s proliferatives and migratory state on the course of inducing stem cells and vessel sprouting for vascular growth factor’s secretion or new vessel formation. There is a need for more exploration of the mechanism and Chinese herbal medicines effective molecular targets.^90^

Furthermore, a notable effect in a PAD mouse model was indicated by VEGF-mimicking peptide. It helps perfusion restoration and considerable improvement in protection and function of animal’s tissue structure with induced acute limb ischemia.^91^

Furthermore, adeno-associated viral vectors showing Ang1 and VEGF were coinjected into the porcine myocardium at several different sites. Results showed that the VEGF/Ang1 group had a better cardiac function and myocardial perfusion in comparison with the controls; also, the VEGF/Ang1 group had higher vascular density and more proliferating cardiomyocytes.^92^ The miR-27b mimicry *in vivo* effects in the mouse model of MI and critical limb ischemia was investigated. The results obtained showed that miR-27b mimic can have suitable effects like increased vascularization and ejection fraction, and fibrosis was also diminished.^93^ Moreover, the miR-126-3p in vitro potential for angiogenesis in chronic ischemia was verified. Cao et al adopted the HUVECs and the ultrasound-targeted microbubble destruction technique was employed for miR-126-3p delivery. Briefly, tissue perfusion, vessel density, and arteriolar formation improved.^94^ Ang1 and VEGF sustained dual delivery could result in an increase in cardiac muscle preservation, repair, myocardial function, and arteriogenesis in the acute MI rat’s model.^95^ In vitro research indicated that PDGF fibrin matrix-conjugated could enhance tissue regeneration in the ischemic flap model. This effect happens by angiogenesis stimulation and perfusion and cardiac function improvement.^96^ Furthermore, Sonnenberg et al delivered an HGF fragment in an extracellular matrix-derived hydrogel to consider the beneficial and potential HGF effects in the MI treatment. Fibrotic marker’s down-regulation induced by HGF-f, Compared to the control, resulted in enhanced arteriole density, and improved myocardial function in the MI rat’s model.^97^ Besides, *in vitro* and* in vivo* research indicated that VEGF/Ang2 could increase the formation of microvascular and vessel maturation in the blood vessel formation subcutaneous model.^98^ Paul et al in another study designed a new injectable Hydrogel to deliver the VEGF165 gene and graphene oxide nano complex for myocardial therapy. The therapeutic hydrogel intramyocardial injection in the acute MI rat’s model indicated reduced scar area and notable enhancement and development in the myocardial capillary density.^99^

## Disadvantages of angiogenesis and overview of angiogenesis inhibitors

**Figure 3:**
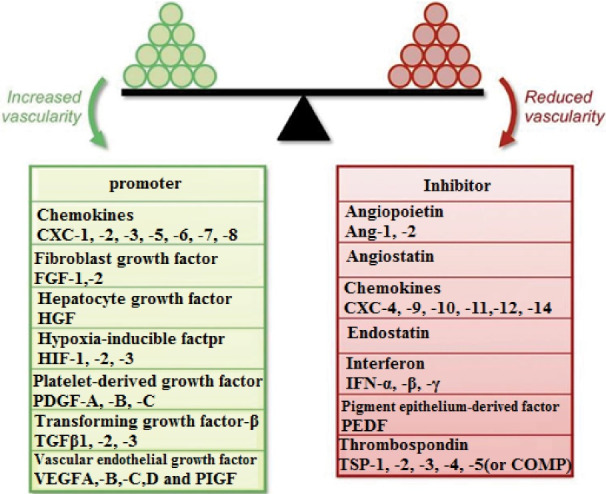
The representation of angiogenic balance showing the different angiogenic inducers and inhibitors ^103^

The process of angiogenesis is the consequence of a balance and equivalence between inhibiting and stimulating factors that the alteration and variation in the equivalence of anti-angiogenic and pro-angiogenic molecules defined as “angiogenic switch”.^100^ Interleukin-10, interleukin-12, Ang2, angiotensin, endostatin are some of the important endogenous regulators of angiogenesis (Figure 3).^101-103^

As mentioned earlier, angiogenesis, as it plays an important role in the regeneration of body tissues (eg, wound healing), also has significant effects on cancer growth and progression such a way that neovascularization (induction of angiogenesis) is one of the notable strategies that the tumor uses to continue its metastasis and growth. Previous studies have shown that angiogenesis plays a more crucial and important role in tumors larger than 1–2 mm in size, in such a way that these tumors cannot grow without angiogenesis.^104,105^ Because of the key role of angiogenesis in tumor growth, inhibiting angiogenesis is one of the important options in the treatment of cancer, regardless of the tissue of origin, and therapeutic inhibition of tumor angiogenesis is one of the most heavily studied areas of cancer research. In recent years, researchers have found numerous agents of angiogenesis inhibitors, some of which have completed their pre-clinical and clinical studies and have shown acceptable effects on inhibiting tumor growth as one of the promising ways to treat cancer.^104^ Some of the FDA approved angiogenesis inhibitors and their indications are provided in Table 3.^106^ The main purpose of treatment with angiogenesis inhibitors in the treatment of cancer is the suppression of metastasis in high-risk patients and the prevention of recurrence in high-staging patients. The success rate of this treatment is not precise and depends on various factors such as the type of cancer, time of diagnosis, and stage of the disease. These drugs may be more effective in treatment regimens combined with other medicine.^107^

**Table 3:** Some of the FDA approved angiogenesis inhibitors and their indications.

**Drug**	**Brand name**	**Indications**	**Mechanism**
Bevacizumab	Avastin, Mvasi	Cervical cancer, colorectal cancer, fallopian tube, ovarian (epithelial) or primary peritoneal cancer, non-small cell lung cancer, renal cell carcinoma, nonsquamous, glioblastoma, diabetic macular edema	Monoclonal anti-VEGF antibody (VEGF-A)
Aflibercept	Zaltrap, Eylea	Colorectal cancer age-related macular degeneration, diabetic retinopathy, diabetic macular edema, macular edema	Recombinant fusion VEGF protein (PLGF, VEGF-A, VEGF-B)
Ramucirumab	Cyramza	Colorectal cancer, gastric cancer, hepatocellular carcinoma, non-small cell lung cancer	Monoclonal anti-VEGFR2 antibody
Sorafenib	NexAVAR	Hepatocellular cancer, renal cell cancer, thyroid cancer	Multitargeted tyrosine kinase inhibitor (PDGFRs, VEGFRs, RAF, KIT, FLT3, RET)
Sunitinib	Sutent	Gastrointestinal stromal tumor, pancreatic neuroendocrine tumors, renal cell carcinoma	Multitargeted tyrosine kinase inhibitor (VEGFRs, FLT3, PDGFRs, CSF1R, RET)
Pazopanib	Votrient	Renal cell carcinoma, soft tissue sarcoma	Multitargeted tyrosine kinase inhibitor (PDGFRs, FGFR1–2, c-Kit, VEGFRs)
Axitinib	Inlyta	Renal cell carcinoma	Multitargeted tyrosine kinase inhibitor (PDGFRs, c-Kit, VEGFRs)
Vandetanib	Caprelsa	Medullary thyroid cancer	Multitargeted tyrosine kinase inhibitor (VEGFRs, RET, EGFR)
Regorafenib	Stivarga	Colorectal cancer, gastrointestinal stromal tumors, hepatocellular carcinoma	Multitargeted tyrosine kinase inhibitor (VEGFRs, FGFRs, TIE2, PDGFRs, KIT, RET, RAF)
Lenvatinib	Lenvima	Endometrial carcinoma, hepatocellular carcinoma, renal cell carcinoma, thyroid cancer	Multitargeted tyrosine kinase inhibitor (VEGFRs, FGFRs, PDGFRa, RET, c-Kit)
Cabozantinib	Cabometyx	thyroid cancer, Hepatocellular carcinoma, renal cell carcinoma	(VEGFRs, Tie2, cMet, AXL)
Ranibizumab	Lucentis	macular degeneration, Diabetic macular edema, diabetic retinopathy, macular edema, myopic choroidal neovascularization	Monoclonal anti-VEGF antibody (VEGF-A)
Pegaptanib	Macugen	Macular degeneration (neovascular age-related)	An aptamer that bind to VEGF

The role and impression of angiogenesis in atherosclerosis and other CVDs have known as an enigmatic unresolved issue. Although, the angiogenic cytokine therapy considered as an engrossing option for increasing the arterioprotective activities of the endothelium, and treating ischemic heart disease; In the results of conflicting studies, neovascularization has been introduced as one of the effective factors in the growth and rupture of atherosclerotic lesions and neointima formation. Howbeit there is compelling evidence that the development of human atherosclerotic plaques, requires and accompanies the process of angiogenesis and constitution of novel micro vessels within these plaques, but the proof of whether angiogenesis and neovascularization can cause plaque or lead to lesion growth and its progression are difficult and ambiguous.^108^ Rheumatoid arthritis (RA) is another case that can aggravate via angiogenesis. It is a chronic autoimmune disease that causes functional loss and progressive articular damage, It is the most common inflammatory arthropathy. The pathophysiology of inflammatory diseases such as RA and solid tumors are remarkably resemblance. In both cases, hypoxia and cytokines play important roles in angiogenesis.^109^ Previous studies have underscored the undeniable role of angiogenesis in the progression of RA disease and have also suggested that inhibition of synovial angiogenesis could supply an important promising therapeutic strategy.^110^

Furthermore inappropriate and excessive or unnecessary growth of blood vessels one of the most important reasons in ocular disorders such as AMD and diabetic retinopathy that performs a major role in their pathology. AMD is a multifactorial disease affected by a composition of genetic variants and environmental factors. In this disorder, the macular region of the retina is damaged, causing serious central vision loss, it’s one of the leading causes of irreversible blindness in people older than 50 years.^107,111^ Anti-VEGF agents, as an antiangiogenic strategy, showed significant effects and notable efficacy in patients and could considerably improve the central vision.^111^ Pegaptanib sodium (Macugen) is the first anti-VEGF drug that was approved to treat neovascular ADM.^111^ Also, ranibizumab is the new VEGF Inhibitor that has FDA approval for treatment of neovascular (wet) AMD, diabetic macular edema, diabetic retinopathy and myopic choroidal neovascularization. Similarly, bevacizumab is another monoclonal anti-VEGF antibody (VEGF-A), that is intravenously administered to treat ADM.^111,112^ Studies have shown that bevacizumab had significant effects on improving the status of patients with ADM and no significant adverse effects were observed.^113,114^ Aflibercept (Eylea) is a potent recombinant fusion VEGF protein (PLGF, VEGF-A, VEGF-B) that is used to treat colorectal cancer, AMD, diabetic macular edema, diabetic retinopathy, and macular edema. The safety and efficacy of every-2-month and monthly dosing of intravitreal aflibercept injection in wet AMD compared monthly ranibizumab was investigated. All of the aflibercept groups showed the same effects as the monthly injections of ranibizumab and also reported similar side effects.^111,115^

## Conclusion and future perspectives

Because of CVDs’ high prevalence and their economic consequences for countries, more clinical and basic trials and better-designed studies for the development of effective and novel therapeutic approaches are a must. Recent stem cell biology progress has attracted attention to the therapies that can increase the patients’ regeneration potential. Today, we know that a stem cell can be obtained by reprogramming any cell type, and stem cells can produce an infinite number of different new cell types such as endothelial cells, cardiomyocytes, and neurons. Employing stem cell biology through clinical efforts is started and in progress to be used for heart failure. Regenerative biology must strive to overcome technical and economic barriers to pave the way for heart disease’s realistic treatments. Finally, it will make profound progress in the field of heart diseases. Because of CVDs’ high prevalence and their economic consequences for countries, more clinical and basic trials and better-designed studies for the development of effective and novel therapeutic approaches are a must. Recent stem cell biology progress has attracted attention to the therapies that can increase the patients’ regeneration potential. Today, we know that a stem cell can be obtained by reprogramming any cell type, and stem cells can produce an infinite number of different new cell types such as endothelial cells, cardiomyocytes, and neurons. Employing stem cell biology through clinical efforts is started and in progress to be used for heart failure. Regenerative biology must strive to overcome technical and economic barriers to pave the way for heart disease’s realistic treatments. Finally, it will make profound progress in the field of heart diseases.^116^

## Ethical issues

 Not applicable.

## Conflicts of interest

 None declared.
